# Transcriptional Analysis of Distant Signaling Induced by Insect Elicitors and Mechanical Wounding in *Zea mays*

**DOI:** 10.1371/journal.pone.0034855

**Published:** 2012-04-12

**Authors:** Jurgen Engelberth, Claudia Fabiola Contreras, Sriram Viswanathan

**Affiliations:** Department of Biology, University of Texas at San Antonio, San Antonio, Texas, United States of America; Kansas State University, United States of America

## Abstract

When plants are under insect herbivore attack defensive measures are activated not only locally, but also in distant and systemic tissues. While insect elicitors (IE) abundant in the oral secretions of the attacking herbivore are essential in the regulation of induced defenses, little is known about their effects on systemic defense signaling in maize (*Zea mays*). The goal of this study was therefore to identify genetic markers that can be used to further characterize local and systemic signaling events induced by IE or mechanical wounding (MW). We selected genes for this study based on their putative involvement in signaling (allene oxide synthase), regulation of gene expression (transcription factor MYC7), and in direct defenses (ribosome inactivating protein) and analyzed their expression in different sections of the treated leaf as well as in systemic parts of the same plant. We found the most significant transcript accumulation of the selected genes after treatment with insect elicitors in those parts with increased JA levels. Additionally, treatment with IE did also induce the accumulation of MYC7 transcripts in basal parts of the treated leaf and systemically. MW, in contrast, did induce RIP and AOS only locally, but not MYC7. This local suppression of MYC7 was further studied by adding glutathione (GSH) as an electron donor to MW plants to quench putative α, β-unsaturated carbonyls, which build up to significant levels around the damage site. Indeed, GSH-treated MW plants accumulated MYC7 at the damage site and also produced more volatiles, suggesting a putative redox-regulatory element being involved in the suppression of MYC7. The results presented herein provide evidence for the specific induction of distant signaling events triggered by IE, most likely through electric signaling. Additionally, a putative role for MW-induced α, β-unsaturated carbonyls in the transcriptional regulation of defense genes was discovered.

## Introduction

Plants in natural settings and agriculture are constantly exposed to a multitude of biotic stresses mainly caused by pathogen infection and insect herbivore attack. In order to fend off these threats plants have developed a diverse array of defensive strategies, all aiming toward the reduction of damage. For insect herbivory signaling mechanisms that activate anti-herbivore defenses include the recognition of movement, mechanical damage, and compounds in the oral secretions of insect herbivores, all leading to the production of inducible defenses like toxic secondary metabolites and proteins that inhibit the digestion of nutrients in the insect gut system [1]–[3].

Insect elicitors (IE) abundant in the oral secretions of many insect herbivores were shown to induce defense responses in maize seedlings that were comparable to those observed after real caterpillar damage [4], [5]. Further studies led to the discovery of fatty acid-amino acid conjugates as the major elicitors, in particular volicitin, which was named for its capacity to induce volatile release from maize [6]. Volicitin is composed of linolenic acid, which conjugated to glutamine. Furthermore, the linolenic acid portion is hydroxylated in position 17. Further analyses of insect oral secretions revealed the abundance of related compounds that also exhibit elicitor activities like linolenoyl-glutamine and linolenoyl-gluatmate [7] and were also found in crickets and fruit flies [7]–[9]. Other IE that have been identified in recent years were the inceptins, a peptide elicitor isolated from *Spodoptera frugiperda* [10], and the caeliferins from grasshopper *Schistocerca americana* [11]. However, in a comprehensive study by Schmelz and coworkers [4] it was shown that volicitin and *N*-linolenoyl-glutamine had the highest biological activity in maize seedlings when analyzed as accumulation of jasmonic acid (JA).

Indeed, most of the countermeasures plants activate when under insect herbivore attack are signaled through JA [2], [3]. The biosynthesis of JA begins in the chloroplast by incorporating molecular oxygen into α-linolenic acid by a 13-lipoxygenase (LOX), resulting in 13-hydroperoxy-linolenic acid (13-HPLA). 13-HPLA is then converted by allene oxide synthase (AOS) and allene oxide cyclases (AOC) to 9*S*, 13*S*-12-oxo phytodienoic acid (or *cis*-OPDA). *Cis*-OPDA then undergoes 3 cycles of β-oxidation eventually yielding (+)- *iso* JA (or *cis* (*epi*) JA). However, JA needs to be conjugated to an amino acid, for example isoleucine (Ile) resulting in the bioactive JA-Ile [12], [13]. JA-Ile binds to its receptor COI1, which is an essential part of a SCF-protein complex (SCF^COI1^). The target for this complex is a JAZ protein, which acts as a suppressor of JA-activated transcription factors [14], [15]. The binding of the SCF^COI1-JA-Ile^-protein complex to JAZ leads to the polyubiquitination and subsequent degradation of the JAZ-repressor in a 26S-proteasome. Transcription factors like MYC2 then initiate the transcription of typical JA-inducible genes [14]–[16]. Functional orthologs of the activator MYC2, its suppressor JAZ1, and corresponding *cis*-regulatory elements have been identified in *Arabidopsis*, tomato, tobacco, and periwinkle [17], [18], and appear to be quite conserved. But while MYC2 appears to be a major regulator of JA mediated responses and multiple functions for this transcription factor have been described in the literature, little is known about this regulatory mechanism in maize.

The induction of defenses, which are mostly regulated by JA, does not seem to be limited to the area of actual damage. Often, within minutes or hours many inducible defenses are also activated distant undamaged parts of the plant and aid to the protective measures plants undertake to fend off insect herbivores [19]–[24]. Systemic signaling studies have focused on tomato, where wounded leaves synthesize systemin, an 18-amino acid peptide derived from prosystemin. Systemin leaves the phloem parenchyma cells and travels to companion cells where it presumably binds to a systemin receptor in the cell surface. This triggers signaling cascades resulting in the accumulation of JA, which then gets transported in the phloem to other tissues where defense genes coding for protease inhibitors get activated [20], [25], [26]. But although the systemic response has been well studied in tomato, polypeptide-mediated systemic signaling as well as corresponding receptors have not been found in plants other than Solanaceae after herbivory, suggesting other signaling mechanisms in different plant species. In this context IE seem to play an important role in long distance signaling. In *Nicotiana attenuata* IE elicited a rapid activation of MAPK activity in undamaged areas of the same leaf [21]. In maize treatment with IE induced JA in distal tissues of the damaged leaf, but no increase in JA was found in basal [27] or systemic tissue (data not shown). However, evidence for the existence of systemic signaling in maize was provided by gene expression analyses in undamaged leaves. For example, an increase in the accumulation of a sesquiterpene cyclase in systemic leaves after treatment with IE has been described [22]. Likewise, a lipoxygenase (LOX5) was found to be inducible in systemic parts of the plant [23]. However, in all these cases little to nothing is known about the actual signaling pathway that enables the plant to alert distant tissue and regulate gene expression. In *Arabidopsis*, systemic signaling has been analyzed in response to mechanical wounding (MW) [24]. It was shown that JA-Ile accumulation increased in damaged leaves as well as undamaged systemic leaves within minutes after treatment and that JA and JA-Ile were not transported from the wounded to the systemic tissue, but synthesized *de-novo* in the respective leaf. This strongly suggests the existence of a mobile signal other than JA itself [24]. Such a signal was described by [28] and termed system potential due to its capacity to provide long-distance apoplastic signaling in response to MW and fusicoccin, an activator of the plasma membrane H^+^-ATPase. In contrast to action or variation potentials, ion movements were observed for Ca^2+^, K^+^, H^+^, and Cl^−^ after the onset of the voltage change. Further proof for this type of signaling was provided by using alamethicin, a channel-forming peptide with a preference for H^+^, which was shown to produce a similar kind of long-distance signal [29].

From all these studies it is obvious that distant signaling occurs in plants in response to MW or through the activity of IE. However, since past reports did often not clearly distinguish between MW and IE treatment [21], [22], the study presented herein was initiated to carefully compare the effects of these treatments on local and systemic signaling in maize as our model plant. Previously, we analyzed JA accumulation in different areas of maize leaves that were either mechanically damaged or treated with IE [27]. We found that MW alone only induced accumulation at the immediate site of damage, whereas IE also induced JA accumulation in distant (leaf upwards) tissues. For both treatments no accumulation of JA was found basipetal from the damage site. However, since that study was not accompanied by gene expression study, which is often more sensitive than other analytical techniques like hormone analyses, we decided to perform a transcriptional analysis to gain more insight into distant signaling events after MW and application of IE. Based on our hypothesis that IE are an essential for the activation of distant and systemic anti-herbivore defense regulation, we set out to compare MW with IE treatment (here: *N*-linolenoy-glutamine) with regard to differential gene expression in various areas of the treated plant. We selected genes for this experiment based on their potential involvement in signaling through biosynthesis of JA (allene oxide synthase, AOS), regulation of gene expression (transcription factor MYC7), and in direct defenses (ribosome inactivating protein, RIP). The goal of the experiments described herein was to determine the reliance of the selected genes as markers for the treatments chosen for this study and to gain more insight into their potential role in regulation and execution of anti-herbivore defenses in maize.

## Results

### Distal, local, and basal response

The effects of distant signaling on gene expression were studied by analyzing transcript accumulation of a set of genes involved in the defense response of maize. Previous studies in maize have measured JA levels in response to IE and MW in local, distal and basal segments of the treated maize leaves [27]. Based on these studies a time course was established for the experiments presented herein. We treated *Zea mays* var. Kandy Korn seedlings with IE or MW and measured transcript expression in distal, local and basal sections of the treated leaf as well as in systemic tissues by using a quantitative PCR approach to allow for the detection of small levels of the respective gene transcript.

**Figure 1:**
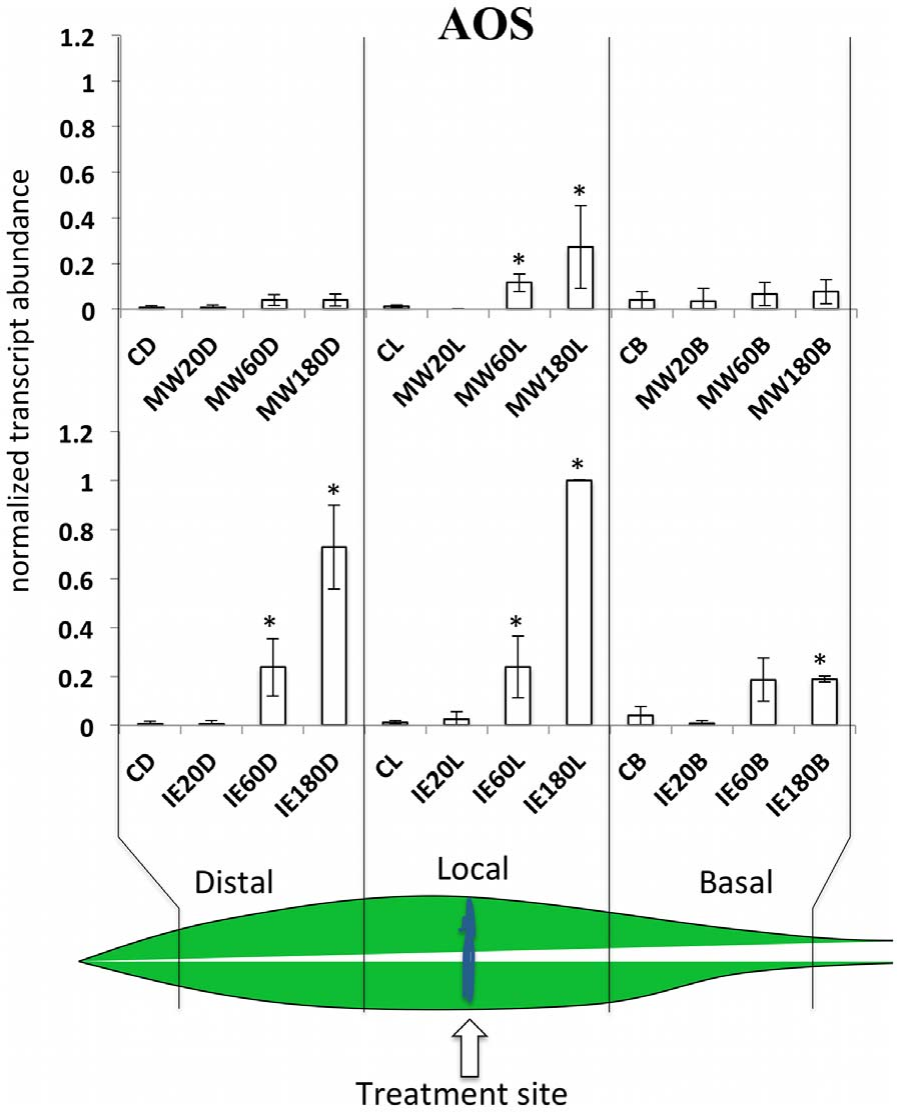
Mechanical wounding (MW) and insect elicitor (IE) induced within-leaf expression of AOS. Transcript accumulation was measured after MW and IE treatment in distal (leaf upward), local (damage site), and basal segments of the second leaf at different time points. Upper panel shows the response to MW. Lower panel shows results for IE. Gene expression is shown as PCR/GapC product. Data was normalized. All experiments have been performed with at least three biological replicates. A schematic maize leaf has been added to demonstrate the experimental setup. Designation of treatments is as follows: C, control, D, distal; L, local; B, basal; MW, mechanical wounding; IE, insect elicitor (here: *N*-linolenoyl-gluatamine); 20, 60, 180, time after treatment in minutes. A Student t test was used for proof of significance (*, P≤0.05) compared with the respective control.

AOS is generally considered to be the bottleneck enzyme for the biosynthesis of JA [30], [31]. Like all genes for this pathway it has also been shown to be inducible by JA. AOS transcript accumulation was found to be significantly increased locally for both, MW and IE treatment (Figure 1). For IE treatment a significant increase was also found in the distal region of the leaf and, to some extent in the basal region. With the exception of the basal region after IE treatment AOS transcript accumulation correlated well with the results in [27], which showed that JA also increases in those leaf areas.

**Figure 2:**
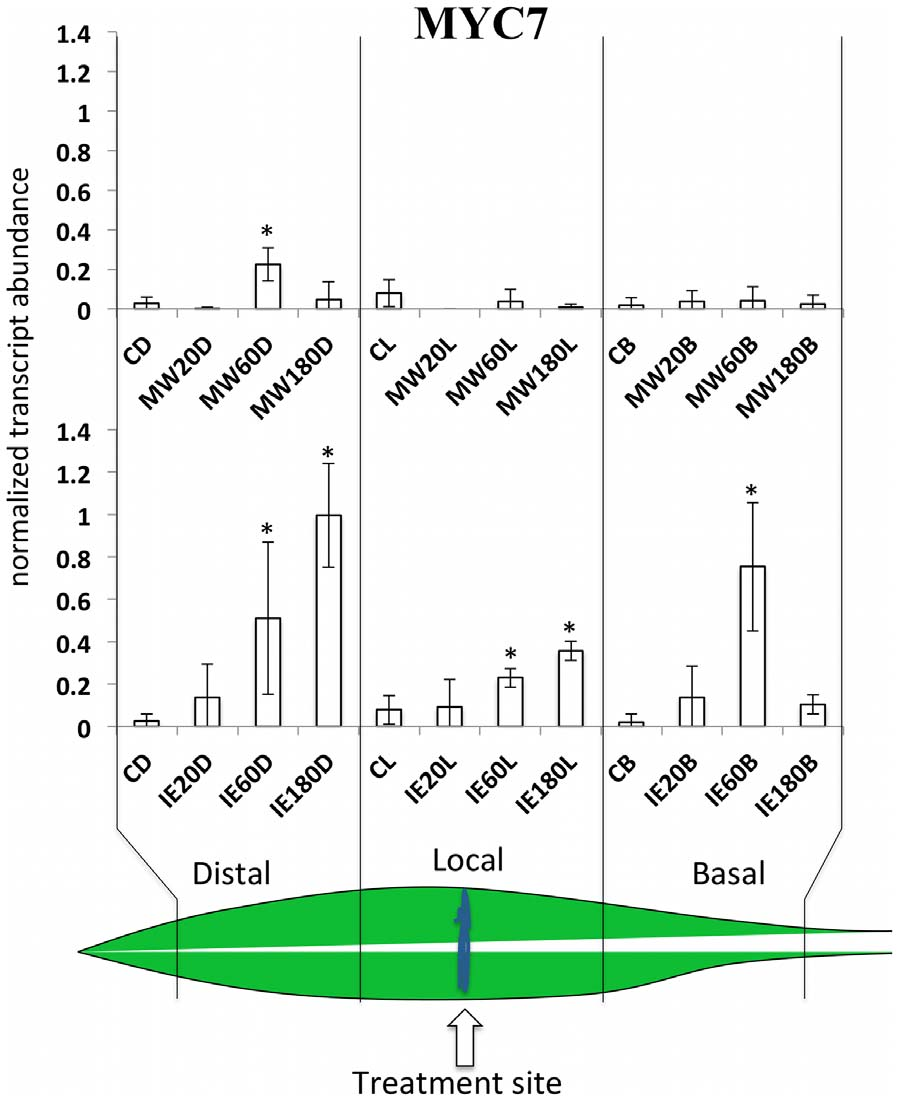
Mechanical wounding (MW) and insect elicitor (IE) induced within-leaf expression of MYC7. Transcript accumulation was measured after MW and IE treatment in distal (leaf upward), local (damage site), and basal segments of the second leaf at different time points. Upper panel shows the response to MW. Lower panel shows results for IE. Gene expression is shown as PCR/GapC product. Data was normalized. All experiments have been performed with at least three biological replicates. A schematic maize leaf has been added to demonstrate the experimental setup. Designation of treatments is as follows: C, control, D, distal; L, local; B, basal; MW, mechanical wounding; IE, insect elicitor (here: *N*-linolenoyl-gluatamine); 20, 60, 180, time after treatment in minutes. A Student t test was used for proof of significance (*, P≤0.05) compared with the respective control.

MYC7 is a putative ortholog of the *Arabidopsis* MYC2 transcription factor, which plays an essential role in the regulation of JA-induced defense responses in this plant and others [17], [18]. We found MYC7 upregulated mainly in those leaves that were treated with IE (Figure 2). There, all segments showed significant transcript increases including the basal segment. For MW treatment we found a small but significant increase of MYC7 expression in the distal section of the leaf, whereas in the local and basal section no transcript accumulation could be detected. From this data it was obvious that MYC7 expression did not correlate with JA accumulation in the respective segment of the leaf. For example, no increases in free JA were ever detected in the distal leaf segment after MW [27]. Likewise, for IE and MW treatment no elevated JA levels were ever found in the basal section of the treated leaf. Most unusual, however, is the fact that after MW in the local section no MYC7 expression could be detected since this is the only area where JA accumulates after this treatment. This strongly suggested that other mechanisms may regulate the activity of MYC7 expression in these areas, which appears to be independent of JA.

**Figure 3:**
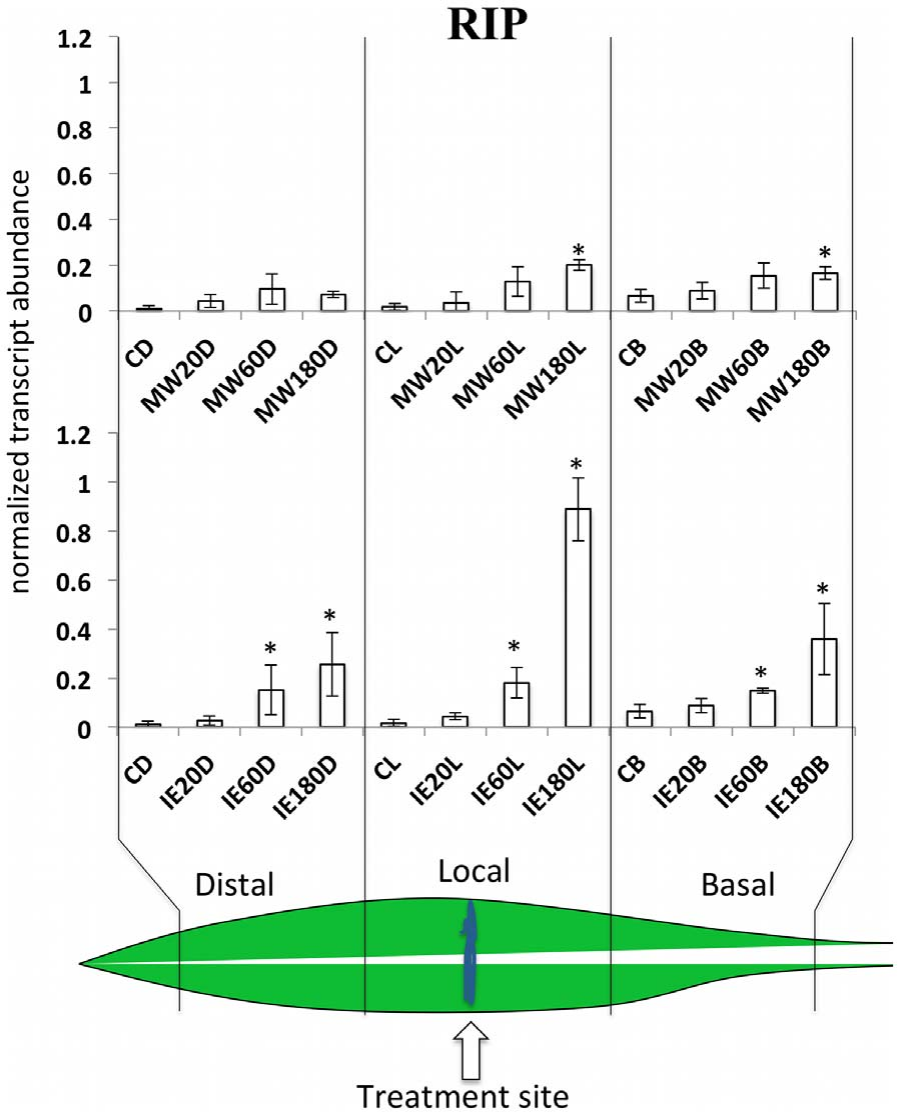
Mechanical wounding (MW) and insect elicitor (IE) induced within-leaf expression of RIP. Transcript accumulation was measured after MW and IE treatment in distal (leaf upward), local (damage site), and basal segments of the second leaf at different time points. Upper panel shows the response to MW. Lower panel shows results for IE. Gene expression is shown as PCR/GapC product. Data was normalized. All experiments have been performed with at least three biological replicates. A schematic maize leaf has been added to demonstrate the experimental setup. Designation of treatments is as follows: C, control, D, distal; L, local; B, basal; MW, mechanical wounding; IE, insect elicitor (here: *N*-linolenoyl-gluatamine); 20, 60, 180, time after treatment in minutes. A Student t test was used for proof of significance (*, P≤0.05) compared with the respective control.

**Figure 4:**
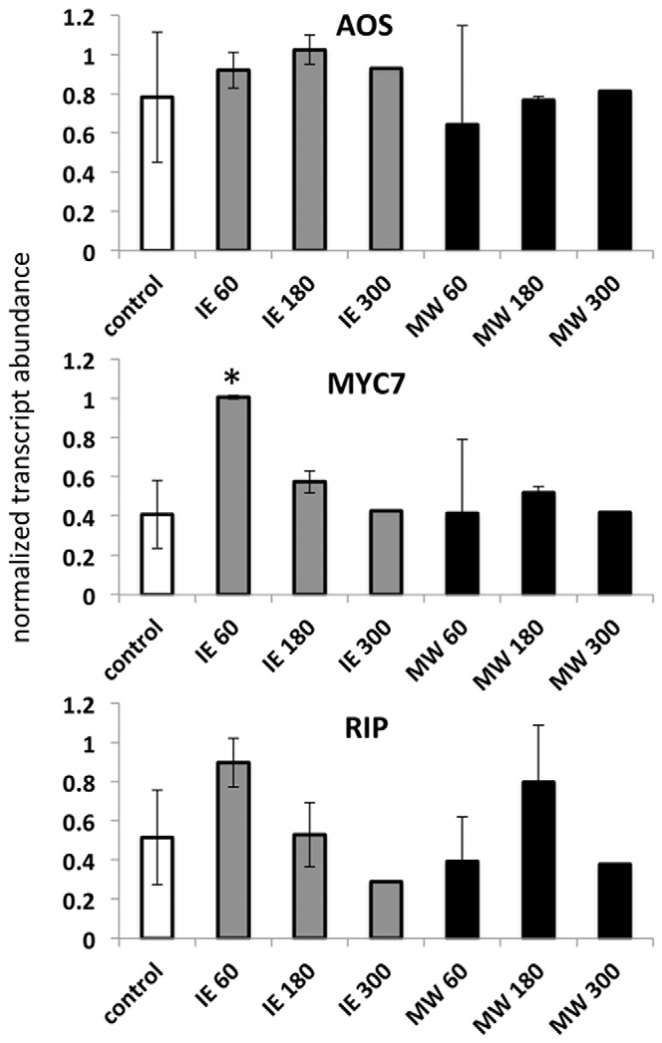
Systemic expression of AOS, MYC7, and RIP in response to mechanical wounding (MW) and application of insect elicitors (IE). Transcript accumulation was measured after MW and IE treatment in systemic leaves at different time points. Gene expression is shown as PCR/GapC product. Data was normalized. All experiments have been performed with at least three biological replicates. Designation of treatments is as follows: MW, mechanical wounding; IE, insect elicitor (here: *N*-linolenoyl-gluatamine); 60, 180, 300 time after treatment in minutes. A Student t test was used for proof of significance (*, P≤0.05) compared with the respective control.

RIP is a defense gene that has previously been demonstrated to significantly affect herbivore performance on maize plants [32]–[34]. RIP has been shown to be inducible by mechanical wounding and insect herbivory. After MW we found small but significant increases of RIP transcripts in the local and basal region of the treated leaf, whereas in the distal part of the same leaf no increase could be detected (Figure 3). For the IE-treated plants we found significant increases in all three segments with the highest accumulation in the local segment. As for the other genes tested in this study RIP transcript accumulation did not correlated with previously reported JA accumulation [27]. In particular, the increased transcript accumulation in the basal part of the IE-treated leaf strongly suggested a JA-independent mechanism that signals herbivory in a basipetal manner and may ultimately reach other, systemic parts of the plant. Therefore, we also tested AOS, MYC7, and RIP for transcript accumulation in a systemic leaf after treatment with IE and MW (Figure 4). For MW we could not detect any transcript accumulation of the selected genes in a systemic leaf within the chosen time frame (up to 5 h). Likewise, for IE treatment no increases could be found for RIP and AOS transcripts. However, MYC7 transcripts increased within 60 min after treatment with IE. As described for the basal section of the treated leaves above, we were not able to detect any increases in JA in the systemic leaf in response to IE. Therefore, other signaling mechanisms may be involved in the activation of MYC7 in systemic signaling.

### JA-Ile and Glutathione response

**Figure 5:**
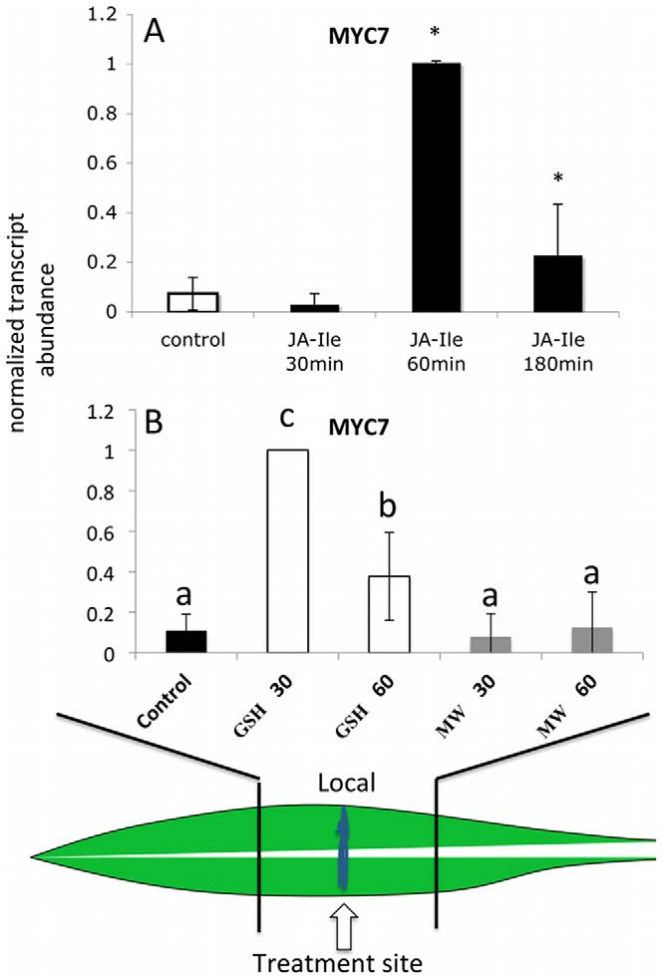
Effects of Jasmonyl-Isoleucine (JA-Ile) and Glutathion (GSH) on MYC7 transcript accumulation after mechanical wounding. JA-Ile (A) and GSH (B) were added to wounding sites as described in Material and Methods and transcript accumulation of MYC7 was measured at different time points as indicated. Data was normalized. All experiments have been performed with at least three biological replicates. A Student t test was used for proof of significance (*, P≤0.05) compared with the respective control (A). For (B) different letters (a–c) represent significant differences for MYC7 transcript accumulation (All ANOVA P values<0.01 with Tukey test corrections for multiple comparisons; P<0.05).

Since MYC7 expression was not increased in the local response after MW albeit the fact that JA is significantly upregulated in this area, we designed several experiments to gain more insight into putative mechanisms that may suppress the expression of this transcription factor. It was first established that the biologically active derivative of JA, JA-Isoleucine (JA-Ile) was able to induce MYC7 expression. We found that MYC7 transcripts were significantly upregulated by JA-Ile (Figure 5A) when applied directly to the MW site. Therefore, we hypothesized that locally after MW, MYC7 expression may be blocked by some mechanism that is specific for the wound response. From previous studies with maize it was evident that besides JA also one of its precursors, 12-oxo-phytodienoic acid (OPDA) accumulates specifically at the side of damage [27]. This suggested that OPDA may act locally not only as a biosynthetic precursor of JA but also as regulator of a subset of distinct responses. OPDA is characterized by a cyclopentenone forming an α, β-unsaturated carbonyl. As such it was proposed repeatedly to form Michael adducts with free –SH and –NH_2_ groups as they occur in proteins. Such addition can alter the configuration of a protein and may render it active or inactive, thereby significantly affecting its performance [35], [36]. In a first approach to test for a possible regulatory effect of OPDA and other α, β-unsaturated carbonyls, i.e. traumatin and E-2-hexenal, we applied glutathione (GSH) to the damage site after MW. GSH is a potential electron donor for Michael additions with its free -SH group and can serve as a potential redox regulator based on its capacity to conjugate to other compounds by forming sulfate esters, Schiff's bases, or Michael adducts [35]–[38]. A high concentration was used to allow for some of the GSH to enter the cells and reduced levels of free OPDA and other α, β-unsaturated carbonyls, which are produced in the vicinity of the damage site. We found that the application of GSH to the wounding site induced MYC7 expression significantly (Figure 5B). Compared to IE-induced activation of MYC7 we found the maximum accumulation for transcripts in the presence of GSH to occur earlier. The highest transcript accumulation was found 30 min after treatment and continued to be still significantly above control level after 60 min. No significant differences in MYC7 accumulation were found for MW-treated plants as well as untreated controls (Figure 5B).

**Figure 6:**
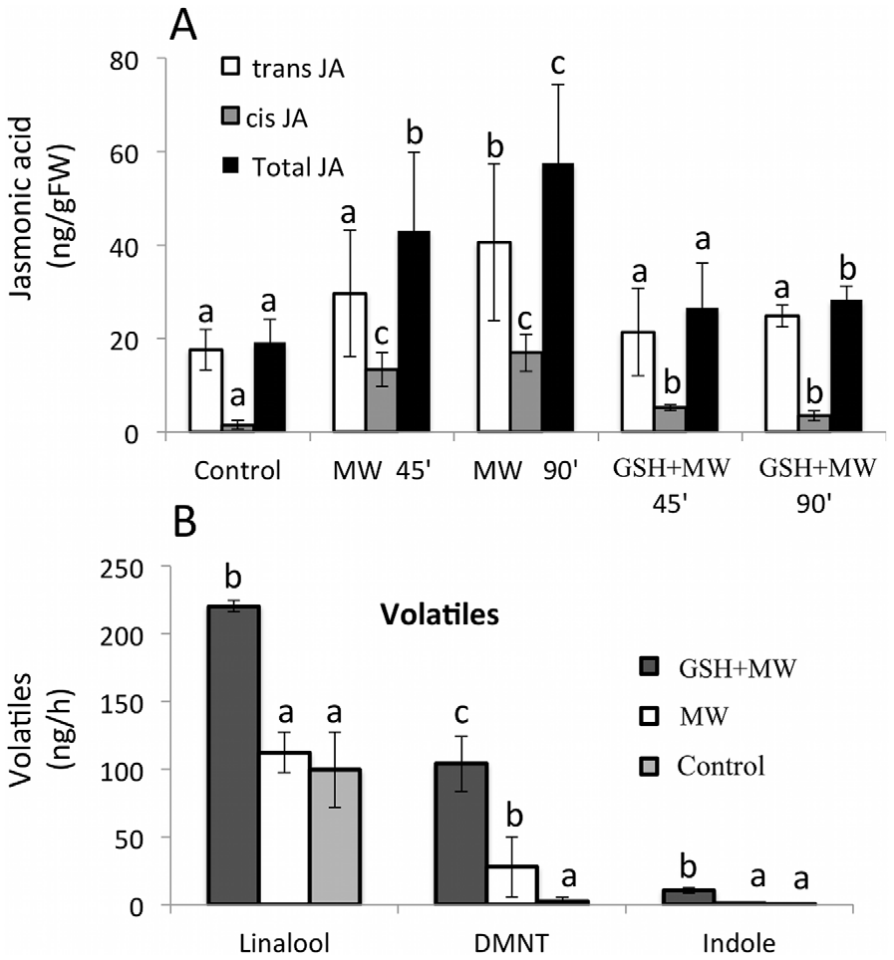
Effects of glutathione (GSH) on jasmonic acid accumulation (A) and volatile release (B). A, GSH was added to the wounding site and Jasmonic acid accumulation (*cis* and *trans*) measured after 45 and 90 min. A Student t test was used for proof of significance between MW and GSH-MW treated plants (*, P≤0.05). B, GSH was added to a wounding site and volatiles release measured after 5 h. All experiments have been performed with at least three biological replicates. Different letters (a–c) represent significant differences for trans JA, cis JA, and total JA, respectively (A) and VOC (B) (All ANOVA P values<0.01 with Tukey test corrections for multiple comparisons; P<0.05). DMNT, 3*E*-4,8-dimethyl-l,3,7-nonatriene.

This increase in MYC7 expression in the presence of GSH supported our hypothesis that local effectors may negatively regulate MYC7 expression. Since OPDA might be a putative target for GSH [37], [38], we also tested for the effects on JA accumulation after MW with and without the addition of GSH. We also found a small but significant reduction in JA accumulation after MW, as it would be expected from this compound due to its potential to covalently bind OPDA (Figure 6A). To test for the consequences of this modified signaling in the wound response of the maize plant we also measured volatiles released from MW damaged plant and compared them to those emitted from MW plant that received GSH. Surprisingly, we found a significant increase in the release of volatiles from GSH-treated MW plants when compared to MW plants (Figure 6B). In particular, linalool, 3*E*-4,8-dimethyl-l,3,7-nonatriene (DMNT), and indole levels were significantly higher in GSH-MW-treated maize seedlings when compared to MW controls. Interestingly, we found only DMNT to be significantly increased in MW plants when compared to undamaged controls.

## Discussion

Herbivory-induced defense responses have been intensively studied in maize, but many aspects of defense-related signaling, in particular those related to distant signaling, still remain unclear. This study attempted to characterize distant signaling events in *Z. mays* in response to MW and induction with IE by analyzing transcript accumulation of selected genes putatively involved in the anti-herbivore defense response.

It has been known for many years that plants under insect herbivore attack not only activate defenses in the immediate vicinity of the damage, but also in other more distant parts of the plant. Often, within minutes to hours, direct defenses become activated in systemic tissues and contribute significantly to the protection of the plant by reducing the nutritional value for the attacking herbivore [2], [3], [5]. Additionally, indirect defenses like the release of volatiles, which enhances the probability of parasitism and predation of the insect herbivore by its natural enemies, are activated in distant tissues and contribute significantly to the overall defense strategy of the attacked plant [1]–[3], [39], [40]. But while the consequences of distant signaling have been described repeatedly in the past, little is known about the mechanisms that regulate these processes.

The best-studied system to date is probably the systemic signaling pathway in tomato mediated by systemin [20]. However, while systemin was long thought to be the mobile signal it is now clear that it is only required to potentiate the wound signal, but does not participate directly in long distance signaling [25]. Rather, JA or one of its derivatives is actively transported through the phloem to systemic tissues, where it activates defenses responses, i.e. the production of proteinase inhibitors In contrast, for *Arabidopsis* it was shown that after mechanical wounding JA-Ile, the bioactive form of JA, accumulated rapidly in systemic leaves [24]. However, in contrast to systemic signaling in tomato, JA or JA-Ile did not seem to be the mobile signal here. The JA-biosynthetic gene *OPR3* was found to be required in the systemic leaf to activate JA-Ile accumulation, but was not necessary in the wounded leaf to initiate the signaling. This strongly suggested a different mechanism for systemic signaling in *Arabidopsis* as compared to tomato since the production of JA in the wounded tissue is not necessary to activate JA-Ile production in systemic leaves. Common to both plants, tomato and *Arabidopsis*, is that mechanical wounding alone is sufficient to induce systemic signaling. In fact, *Arabidopsis* and tomato showed little to no response to the application of insect-derived elicitors [4]. In contrast, IE seem to play an important role not only in the activation of local defense responses but also in the initiation of distant signaling in other plant species including maize [2], [3]. As shown herein, MW did not initiate any significant distant signaling, while the application of IE activated defense-related responses in all above ground-parts of the maize seedling. Interestingly, the activation of these responses did not necessarily correlated with the previously reported JA accumulation in the respective areas of the plant [27]. For example, it was shown that when elicitors were applied to the wounding site, JA accumulated at the damage site and also leaf-upwards to significant levels, while in the basal part of the treated leaf no JA accumulation was ever detected. This may explain some the herein described gene expression, especially those in the local and distal parts, but cannot explain the induction of RIP, AOS, and in particular MYC7 in the basal part of the elicitor treated leaf since no JA accumulation was ever observed in those areas. While we cannot rule out a potential involvement of JA-Ile or other conjugates of JA, we consider this an unlikely option since application of JA-Ile to the wounding did not induce any expression of MYC7 in both, the basal tissue of the treated leaf and a systemic leaf (data not shown) and thus, does not seem to be transported away from the wounding site. Also, RIP and AOS were both found to be up regulated by JA (data not shown), but transcripts of both genes were did not accumulate in systemic tissues. Therefore, a signaling mechanism other than JA has to be postulated. Schittko and coworkers [41] already found that caterpillar feeding generated a very rapid signal, which travelled rapidly from the damage site to other parts of the leaf. They concluded that the speed of the signal was too fast for a chemical to travel through the leaf via the phloem and that compounds from the insect saliva are likely responsible for this effect. Similarly, Wu and coworkers [21] found MAPK activity in undamaged areas of the FAC-treated leaf, further supporting the hypothesis that elicitors are essential for distant signaling. Taken together, all these data strongly point toward electrical signaling as a way by which certain plants alert distant tissues and activate defense responses. Furthermore, insect-derived elicitors are a necessary element for the induction of distant signaling in plants like maize and tobacco. A recent study in broad bean and barley provided evidence that electric signaling might be the mechanism by which these plants facilitate systemic signaling. Zimmermann and co-workers [28] therein described an apoplastic signal termed system potential that moves rapidly through the plant after MW, fusicoccin treatment, and elicitation by alamethicin [29], a channel-forming peptide that has been described previously to induce defense responses.

A marker for FAC-induced distant signaling in maize appears to be MYC7. As described herein, we found transcript accumulation for this gene in all above ground parts of the plant after treatment with elicitor whereas mechanical wounding was without any effect on MYC7 expression. Interestingly, the maximum transcript accumulation for MYC7 in the basal part of the treated leaf and in the systemic leaf correlated in their temporal expression.

MYC7 may be an important regulator of FAC-induced defense responses in maize. Functional orthologs of MYC7 and its suppressor protein JAZ as well as corresponding *cis*-regulatory elements have been identified in various plant species like *Arabidopsis*, tomato, tobacco, and periwinkle, and appear to be quite conserved [17], [18]. All of these orthologs have been show to act as major regulators of JA-mediated defense responses. Most studies to date have focused on MYC2 in *Arabidopsis*. As already described above the transcriptional activity of MYC2 is usually suppressed by JAZ proteins [14], [15]. To remove this suppressor, JA-Ile has to bind to its receptor COI1 in a complex with SCF proteins, which then causes the polyubiquitination and subsequent degradation of the JAZ-repressor in a 26S-proteasome. Once the suppressor is removed MYC2 can initiate the transcription of typical JA-inducible genes [14]–[16], [18] including major defense genes. MYC2 transcripts are rapidly induced after mechanical wounding in *Arabidopsis* and accumulated to maximum levels within minutes correlating well with JA and JA-Ile accumulation in those tissues [16]. The same study also demonstrated that exogenously applied methyl-JA can induce MYC2 expression, strongly suggesting that JA is essential for the induction of MYC2. In this, *Arabidopsis* MYC2 appears to be different from the maize MYC7 gene. For example, we could not detect any significant increase in transcript levels for MYC7 within the early time frame reported for *Arabidopsis*. Also, mechanical wounding did not induce MYC7 despite the fact that JA significantly accumulated in the damaged area [27]. This raised the question of whether JA-Ile as the major bioactive jasmonate would be able to overwrite the local suppression of MYC7 in maize when applied exogenously to the wounding site. Since this was the case (see Figure 5), it has to be assumed that in maize certain factors produced at the wounding site effectively block MYC7 expression. We already showed that for example free OPDA accumulates significantly at the wounding site [27]. While OPDA is an important intermediate of the JA biosynthetic pathway it is also known to exhibit its own biological activity [35], [36], [42]–[45]. Some of this activity has been attributed to the presence of an α-ß unsaturated carbonyl moiety in the molecule, which allows OPDA to covalently bind to proteins and other cellular components, thereby altering their functionality. The same structural feature can also be found in other oxylipins presumably produced at the wounding site like traumatin and *E*-2-hexenal. To test for a potential involvement of these compounds in the suppression of MYC7 gene expression we applied GSH to the damage site. GSH has a free thiol group, which allows it to bind to α-ß unsaturated carbonyls through Michael addition. Additionally, GSH may form conjugates with OPDA *in vivo*, which are transported to the vacuole presumable for degradation [37], [38]. As shown above, GSH treatment caused MYC7 transcript levels to increase significantly and more rapidly after mechanical wounding. Also, as expected, we found significantly reduced JA accumulation in GSH-MW plants since the addition of this compound may interfere with the JA-biosynthetic pathway by binding to OPDA. Surprising however was our finding that GSH application to MW plants significantly increased volatile release from maize seedlings. It is generally accepted that these increases in JA correlate well with increases in volatile production, as it has been observed for elicitor treatment in maize and other plants [4]. However, since JA levels are significantly reduced in GSH-MW plants other factors must have contributed to the increase in volatile release and MYC7 may very well be one of these regulators. Despite interfering with the JA-biosynthetic pathway and other oxylipins featuring an α-ß unsaturated carbonyl, GSH may also directly interact with MYC7 by covalently modifying cysteine residues within the protein. However, this redox-related modification of a MYC transcription factor has not been reported to date and is currently under investigation in our lab.

As shown herein, FAC induce MYC7 transcript accumulation not only in the treated leaf, but also in systemic parts of the plant. This correlates well with previous studies on volatile release in maize after insect herbivory and treatment with IE. Volatiles represent an important part of the overall defense strategy of plants against insect herbivores by attracting natural enemies, reducing oviposition rates, and serving as feeding deterrents [46]–[49]. It has been demonstrated repeatedly that IE treatment induced the release of significantly more volatiles when compared to mechanical damage alone [4], [6], [22], [39], [50], [51]. Turlings and Tumlinson [39] showed that elicitor-treated maize seedling not only produced volatiles locally, but also in systemic leaves. Interestingly, the major volatiles produced in systemic leaves were linalool, indole, and the two homoterpenes, DMNT and (3E, 7E)-4,8,12-trimethyl-1,3,7,11-tridecatetraene (TMTT). While we could not detect any significant release of TMTT in our plants, we found the other three volatiles to be significantly released after treatment with GSH and thus, may be regulated by MYC7. However, to date only two genes, lipoxygenases 5 [23] and a sesquiterpene cyclase [22], have been demonstrated to be upregulated systemically in response to insect herbivory, both of which contain multiple putative MYC binding sites in their respective promoter region, thereby making this transcription factor a likely regulator of these genes. However, our knowledge on systemically up-regulated genes in maize seedling in response to insect herbivory or IE treatment is very limited and we are currently in the process of analyzing systemic gene expression in response to IE on a global scale in an attempt to gain further insights into the regulatory mechanisms.

It seems obvious that MYC7 expression is negatively regulated by yet unknown factors at the wounding site in the absence of IE. While we currently have no evidence as to why maize seedlings block certain signaling pathways after MW alone, we can only speculate about this response. It may simply be a way to save resources since defense is costly, especially for a growing seedling [52]. Only when IE are recognized, which clearly indicates the presence of an insect herbivore, do maize plants appear to initiate the full defensive response, among which the release of volatiles is an important factor. From the data presented herein it seemed likely that MYC7 plays a role in the regulation of genes involved in the production of these volatiles. Not only are they produced locally within a herbivore-damaged or elicitor-treated leaf, but also systemically in yet undamaged areas of the same plant, which correlates well with the expression pattern of MYC7. While there is still little known about the nature of the systemic signal in maize plants it is evident from this and previous studies that insect derived elicitors play an important role in the initiation and specificity of distant signaling.

## Materials and Methods

### Chemicals

Jasmonic acid-isoleucine was purchased from Larodan (Malmö, Sweden). Dihydro jasmonic acid-methyl ester was provided by Bedoukian Research (Danbury, CT, USA) and converted to dihydro jasmonic acid (dhJA) by alkaline hydrolysis. Glutathion (GSH) was purchased from Sigma Aldrich (St. Louis, MO, USA). *N*-Linolenoyl-glutamine was generously provided by Dr. Hans Alborn (USDA, ARS, CMAVE, Gainesville, FL). All solvents used were analytical grade.

### Insect elicitor-induced transcriptional analysis

*Zea mays* (var. Kandy King, J.W. Jung Seed Co. Randolph, WI, USA) plants were grown in soil (Redi Earth Plug and Seedling Mix, Sun Gro) in a growth chamber with a 12 h photoperiod, 60% relative humidity at 26°C for two to three weeks. Light intensity was set at app. 150 µmol m^2^ s^−1^. At this time plants were at the V_2_ stage.

For treatment with N-linolenoyl-glutamine as our model elicitor, the second leaf was scratched as described above and 10 µl of the elicitor solution (100 pmol/µl dissolved in phosphate buffer (50 mM, pH 8), corresponding to 1 spit equivalent) immediately added to the wounding site. To test for distant gene expression after mechanical wounding (MW), maize seedlings were scratched with a razor blade across the midrib in the middle of the second leaf. No buffer was added since MW is usually not associated with liquid deposition. Also, previous studies in our lab showed that application of buffer does not alter the response to MW. Controls consisted of undamaged plants. To analyze within-leaf signaling treated leaves were taken after 20, 60, and 180 minutes and cut into three 2.5 cm segments comprising the distal, local, and basal part of the leaf. At least 3 segments were pooled from each treatment group. To test for systemic signaling, the second leaf was treated as described above for IE and MW and a 5 cm central segment from the third leaf of each treated plant was collected after 60, 180, and 300 minutes. As above, 3 segments were pooled per biological sample. All plant material was shock-frozen in liquid N_2_. Samples were stored at −80°C for later further analysis.

### Effects of JA-Ile and GSH on MYC7 transcript accumulation, volatile release, and JA levels

JA-Ile was prepared to a 300 µM stock solution in 10 mM phosphate buffer (pH 5.7). Plants were treated by scratching the adaxial side going across the midrib and applying 10 µl of the JA-Ile solution (corresponding to 3 nmol JA-Ile per application site). Controls consisted of mechanically wounded plants with 10 µl of buffer as well as untreated plants. The damaged leaves and appropriate section from the undamaged control plants were collected after 30, 60 and 180 minutes, and a 1.5 cm segment (3 segments were pooled per biological replicate) comprising the application site was shock-frozen in liquid N_2_ and stored at −80°C for further processing.

GSH was dissolved in water at a concentration of 10 mM. To test for the effects of GSH on MYC7 transcript accumulation the second leaf of a two-week-old maize seedling was scratched with a razor blade as described above and 20 µl of the GSH solution were immediately added to the wounding site. Mechanically wounded control plants were also scratched, but received 20 µl of water. Additionally, comparable leaf segments of untreated control plants were also analyzed. A 2.5 cm segment from the second leaf comprising the treatment area was collected at 0, 30, and 60 minutes and immediately shock-frozen in liquid N_2_ (3 segments pooled per biological sample) and stored at −80°C for further processing.

To test for the effects of GSH on MW-induced JA accumulation maize seedlings were treated as described above. Leaf segment (2.5 cm) comprising the treatment area were taken at 45 and 90 min and shock frozen in liquid N_2_. Extraction and quantification of JA was performed as described previously [53]. In brief, plant tissues were frozen in liquid N_2_ and about 100 mg of each sample was transferred to 2 ml screw cap tubes containing 1 g Zirmil™ beads (1.1 mm; SEPR Ceramic Beads and Powders, Mountainside, NJ, USA). DhJA (100 ng) was added to the 2 ml tubes prior to sample addition as the internal standard. The samples were mixed with 300 µl of 1-propanol∶H_2_0∶HCl (2∶1∶0.002) and shaken for 30 sec in a Precellys tissue homogenizer ( MO BIO Laboratories, Carlsbad, CA, USA) at 6000 rotations per minute (rpm). Dichloromethane (1 ml) was added to each sample, re-shaken for 10 s in the homogenizer, and centrifuged at 10,000× *g* for 30 sec. The bottom dichloromethane:1-propanol layer was then transferred to a 4 ml glass screw cap vial with care taken to avoid transfer of the upper aqueous layer. The organic phase was evaporated by a constant air-stream and 100 µl of diethyl ether: methanol (9∶1, vol∶vol) added. Carboxylic acids were converted into methyl-esters by the addition of 2 µl of a 2.0 M solution of trimethylsilyldiazomethane in hexane. The vials were then capped, vortexed, and allowed to sit at room temperature for 30 minutes. Excess trimethylsilyldiazomethane was then destroyed by adding an equivalent molar amount of acetic acid to each sample. Volatile metabolites were separated from the complex mixture by vapor phase extraction as described in [53]. The trapped volatiles were then eluted with 150 µl dichloromethane and analyzed by CI-GC/MS [53]. Quantification was based on the internal standard and the fresh weight of the plant material.

To test for the effects of GSH on MW-induced volatile production 2 leaves (2^nd^ and 3^rd^) of a two-week-old maize seedling were scratched in 4 positions each with 2.5 cm between damage sites and 5 µl of the 10 mM GSH solution were immediately added to each damage site. Control MW damaged plants were treated with water and controls consisted of undamaged plants. Plant were incubated for 5 h and then cut at the root base, wrapped in wet tissue paper, and transferred to 200 ml glass cylinders, where VOC were collected for 1 h as described [54], and subsequently analyzed by GC/MS with 3-octen-2-one as internal standard.

### RNA extraction, RT reaction, and semi-quantitative PCR

The pooled leaf material was crushed and mixed with a sterile wooden stick, and approximately 100 mg were taken from each biological replicate for RNA extraction. Total RNA was extracted with the Ultra Clean Plant RNA Isolation Kit (MO BIO Laboratories, Carlsbad, CA, USA) according to the manufacture's instructions with the following modifications. Frozen plant samples were homogenized in 2 ml screw cap FastPrep tubes containing 0.5 g of Zirmil microbeads and 200 µl extraction buffer (PR1) for 20 sec at 6000 in a Pecellys tissue homogenizer (MO BIO Laboratories, Carlsbad, CA, USA). After this initial homogenization step the remaining 800 µl of PR1 were added and the sample again homogenized for 10 sec at 6000 rpm. The extract was then further processed as described in the manufacturer's instructions. DNase treatment was performed with 3.125 µg total RNA with the Turbo DNA free kit (Ambion, Austin, TX, USA). For Reverse transcription 1.525 µg (in 12 µL water) of DNA-free RNA were mixed with 1 µl oligo dT's (100 mM), 1 µl oligo dTs (100 mM), 2 µl RT buffer (10×), 2 µl dNTP's (5 mM each), 1 µl RNase inhibitor (10 U/µl), and 1 µl reverse transcriptase (5 U/µl) (Omniscript kit, Qiagen, Hilden, Germany). The reaction mixture was incubated for 90 min at 37°C. For semi-quantitative analysis of gene expression, the cDNA was diluted (1∶10) and 5 µl from this dilution was used for PCR. Primers were used as follows: AOS forward 5′-GACCGCCTCGACTTCTACTAC-3′, reverse 5′-GAAGAGCAGCTGCTTCACCTT-3′; RIP forward 5′-CCCGTGGAGGACACGGCCTA-3′, reverse 5′-TGTCGCCGTCCTTGCCGAAC-3′; MYC7 forward 5′-GTCTGCTTCCCCGTCGGCAC-3′, reverse 5′-GCGTCGGCGAGCCATAGCAT-3′.

The PCR volume was 20 µl, containing 5× green GoTaq buffer (Promega) (4 µl), MgCl_2_ (25 mM, 1.2 µl), dNTP's (25 mM, 0.32 µl), primers (10 µM, 2 µl), GoTaq polymerase (5 U/µl, 0.2 µl), TagStart antibody (BD Biosciences) (7 mM, 0.2 µl) and antibody dilution buffer (0.8 µl). PCR was performed on a Eppendorf Mastercycler (Eppendorf, Hamburg, Germany). The following program was used for amplification: 95°C for 3 min, then (94°C for 30 sec, 54°C for 30 sec, 68°C for 1 min)×28, then 68°C for 7 min and was well within the linear range for each product. 10 µl of the PCR product were separated on a 2.5% agarose gel for analysis. Ethidium-bromide stained bands were analyzed with a Photodyne documentation system (Photodyne Technologies, Los Angeles, CA, USA) and expression of genes was normalized by comparison with GAPc.

### Statistical analyses

The statistical analysis for determining significant differences between treatments was performed using the software package JMP version 8. At least three biological replicates of all experiments were performed. Where indicated, percentile transformations were used to normalize data. Data were analyzed for significance with *t*-test (p<0.05). ANOVAs were performed on concentrations of JA and induced VOC. Significant treatment effects were investigated when the main effects of the ANOVAs were significant (P<0.05). Where appropriate, Tukey tests were used to correct for multiple comparisons between control and treatment groups. Before statistical analysis, all data were subjected to square root transformation to compensate for elevated variation associated with larger mean values.

## References

[1] Gatehouse JA (2002). Plant resistance towards insect herbivores: a dynamic interaction.. New Phytol.

[2] Howe GA, Jander G (2008). Plant immunity to insect herbivores.. Annu Rev Plant Biol.

[3] Wu J, Baldwin IT (2010). New insights into plant responses to the attack from insect herbivores.. Annu Rev Genet.

[4] Schmelz EA, Engelberth J, Alborn HT, Tumlinson JH, Teal PE (2009). Phytohormone-based activity mapping of insect herbivore-produced elicitors.. Proc Natl Acad Sci USA.

[5] Engelberth J, Baluska F, Witzany G (2012). Plant resistance to insect herbivory..

[6] Alborn HT, Turlings TCJ, Jones TH, Stenhagen G, Loughrin JH (1997). An elicitor of plant volatiles from beet armyworm oral secretion.. Science.

[7] Halitschke R, Schittko U, Pohnert G, Boland W, Baldwin IT (2001). Molecular interactions between the specialist herbivore *Manduca sexta* (Lepidoptera, Sphingidae) and its natural host *Nicotiana attenuata*. III. Fatty acid-amino acid conjugates in herbivore oral secretions are necessary and sufficient for herbivore-specific plant responses.. Plant Physiol.

[8] Pohnert G, Jung V, Haukioja E, Lempa K, Boland W (1999). New fatty acid amides from regurgitant of lepidopteran (Noctuidae, Geometridae) caterpillars.. Tetrahedron.

[9] Spiteller D, Boland W (2003). N-(15,16-Dihydroxylinoleoyl)-glutamine and N-(15,16-epoxylinoleoyl)-glutamine isolated from oral secretions of lepidopteran larvae.. Tetrahedron.

[10] Alborn HT, Hansen TV, Jones TH, Bennett DC, Tumlinson JH (2007). Disulfooxy fatty acids from the American bird grasshopper *Schistocerca americana*, elicitors of plant volatiles.. Proc Natl Acad Sci USA.

[11] Schmelz EA, Carroll MJ, LeClere S, Phipps SM, Meredith J (2006). Fragments of ATP synthase mediate plant perception of insect attack.. Proc Natl Acad Sci USA.

[12] Kang JH, Wang L, Giri A, Baldwin IT (2006). Silencing threonine deaminase and JAR4 in *Nicotiana attenuata* impairs jasmonic acid-isoleucine-mediated defenses against *Manduca sexta*.. Plant Cell.

[13] Staswick PE, Tiryaki I (2004). The oxylipin signal jasmonic acid is activated by an enzyme that conjugates it to isoleucine in *Arabidopsis*.. Plant Cell.

[14] Chini A, Fonseca S, Fernández G, Adie B, Chico JM (2007). The *JAZ* family of repressors is the missing link in jasmonate signalling.. Nature.

[15] Thines B, Katsir L, Melotto M, Niu Y, Mandaokar A (2007). JAZ repressor proteins are targets of the SCFCOI1 complex during jasmonate signalling.. Nature.

[16] Chung HS, Koo AJK, Gao X, Jayanty S, Thines B (2008). Regulation and function of *Arabidopsis JASMONATE ZIM*-domain genes in response to wounding and herbivory.. Plant Physiol.

[17] Pauwels L, Inze D, Goossens A (2009). Jasmonate-inducible gene: what does it mean?. TIPS.

[18] Boter M, Ruiz-Rivero O, Abdeen A, Prat S (2011). Conserved MYC transcription factors play a key role in jasmonate signaling both in tomato and *Arabidopsis*.. Genes & Development.

[19] Havill NP, Raffa KF (1999). Effects of elicitation treatment and genotypic variation on induced resistance in *Populus* : impacts on gypsy moth (Lepidoptera: Lymantriidae) development and feeding behavior.. Oecologia.

[20] Ryan CA (2000). The systemin signaling pathway: differential activation of plant defensive genes.. Biochim Biophys Acta.

[21] Wu J, Hettenhausen C, Meldau S, Baldwin IT (2007). Herbivory rapidly activates MAPK signaling in attacked and unattacked leaf regions but not between leaves of *Nicotiana attenuata*.. Plant Cell.

[22] Shen B, Zheng Z, Dooner HK (2000). A maize sesquiterpene cyclase gene induced by insect herbivory and volicitin: characterization of wild-type and mutant alleles.. Proc Natl Acad Sci USA.

[23] Park YS, Kunze S, Ni X, Feussner I, Kolomiets MV (2010). Comparative molecular and biochemical characterization of segmentally duplicated 9-lipoxygenase genes ZmLOX4 and ZmLOX5 of maize.. Planta.

[24] Koo AJ, Gao X, Jones AD, Howe GA (2009). A rapid wound signal activates the systemic synthesis of bioactive jasmonates in Arabidopsis.. Plant J.

[25] Stratmann JW (2003). Long distance run in the wound response–jasmonic acid is pulling ahead.. Trends Plant Sci.

[26] Degenhardt DC, Refi-Hind S, Stratmann JW, Lincoln DE (2010). Systemin and jasmonic acid regulate constitutive and herbivore-induced systemic volatile emissions in tomato, *Solanum lycopersicum*.. Phytochem.

[27] Engelberth J, Seidl-Adams I, Schultz JC, Tumlinson JH (2007). Insect elicitors and exposure to green leafy volatiles differentially upregulate major octadecanoids and transcripts of 12-oxo phytodienoic acid reductases in *Zea mays*.. MPMI.

[28] Zimmermann MR, Maischak H, Mithofer A, Boland W, Felle HH (2009). System potentials, a novel electrical long-distance apoplastic signal in plants, induced by wounding.. Plant Physiol.

[29] Maischak H, Zimmermann MR, Felle HH, Boland W, Mithoefer A (2010). Alamethicin-induced electrical long distance signaling in plants.. Plant Signal Behav.

[30] Schaller F (2001). Enzymes of the biosynthesis of octadecanoid-derived signaling molecules.. J Exp Bot.

[31] Wasternack C (2007). Jasmonates: An update on biosynthesis, signal transduction and action in plant stress response, growth and development.. Ann Bot.

[32] Stirpe F, Barbieri L, Battelli MG, Falasca AI, Abbondanza A (1986). Bryodin, a ribosome-inactivating protein from the roots of *Bryonia dioica* L. (white bryony).. Biochem J.

[33] Bass HW, Webster C, OBrian GR, Roberts JK, Boston RS (1992). A maize ribosome-inactivating protein is controlled by the transcriptional activator Opaque-2.. Plant Cell.

[34] Bass HW, Krawetz JE, OBrian GR, Zinselmeier C, Habben JE (2004). Maize ribosome-inactivating proteins (RIPs) with distinct expression patterns have similar requirements for proenzyme activation.. J Exp Bot.

[35] Dueckershoff K, Mueller S, Mueller MJ, Reinders J (2008). Impact of cyclopentenone-oxylipins on the proteome of *Arabidopsis thaliana*.. Biochim Biophys Acta.

[36] Böttcher C, Pollmann S (2009). Plant oxylipins: plant responses to 12-oxo-phytodienoic acid are governed by its specific structural and functional properties.. FEBS J.

[37] Ohkama-Ohtsu N, Sasaki-Sekimoto Y, Oikawa A, Jikumaru Y, Shinoda S (2009). 12-oxo-phytodienoic acid-glutathione conjugate is transported into the vacuole in *Arabidopsis*.. Plant Cell Physiol.

[38] Davoine C, Falletti O, Douki T, Iacazio G, Ennar N (2006). Adducts of oxylipin electrophiles to glutathione reflect a 13 specificity of the downstream lipoxygenase pathway in the tobacco hypersensitive response.. Plant Physiol.

[39] Turlings TC, Tumlinson JH (1992). Systemic release of chemical signals by herbivore-injured corn.. Proc Natl Acad Sci USA.

[40] Roese USR, Manukian A, Heath RR, Tumlinson JH (1996). Volatile semiochemicals released from undamaged cotton leaves: a systemic response of living plants to caterpillar damage.. Plant Physiol.

[41] Schittko U, Preston CA, Baldwin IT (2000). Eating the evidence? *Manduca sexta* larvae can not disrupt specific jasmonate induction in *Nicotiana attenuata* by rapid consumption.. Planta.

[42] Stintzi A, Weber H, Reymond P, Browse J, Farmer EE (2001). Plant defense in the absence of jasmonic acid: the role of cyclopentenones.. Proc Natl Acad Sci U S A.

[43] Alméras E, Stolz S, Vollenweider S, Reymond P, Mène-Saffrané L (2003). Reactive electrophile species activate defense gene expression in *Arabidopsis*.. Plant J.

[44] Dave A, Hernández ML, He Z, Andriotis VM, Vaistij FE (2011). 12-oxo-phytodienoic acid accumulation during seed development represses seed germination in *Arabidopsis*.. Plant Cell.

[45] Taki N, Sasaki-Sekimoto Y, Obayashi T, Kikuta A, Kobayashi K (2005). 12-oxo-phytodienoic acid triggers expression of a distinct set of genes and plays a role in wound-induced gene expression in *Arabidopsis*.. Plant Physiol.

[46] Takabayashi J, Dicke M (1996). Plant—carnivore mutualism through herbivore-induced carnivore attractants.. TIPS.

[47] Turlings TCJ, Tumlinson JH, Heath RR, Proveaux AT, Doolittle RE (1991). Isolation and identification of allelochemicals that attract the larval parasitoid *Cotesia marginiventris* (Cresson) to the micro-habitat of one of its hosts.. J Chem Ecol.

[48] De Moraes CM, Mescher MC, Tumlinson JH (2001). Caterpillar-induced nocturnal plant volatiles repel conspecific females.. Nature.

[49] Shiojiri K, Ozawa R, Takabayashi J (2006). Plant volatiles, rather than light, determine the nocturnal behavior of a caterpillar.. PLoS Biol.

[50] Tumlinson JH, Paré PW, Lewis WJ (1999). Plant production of volatile semiochemicals in response to insect-derived elicitors.. Novartis Foundation Symposium.

[51] Baldwin IT (1990). Herbivory simulations in ecological research.. Trends Ecol Evol.

[52] Heil M, Baldwin IT (2002). Fitness costs of induced resistance: emerging experimental support for a slippery concept.. TIPS.

[53] Engelberth MJ, Engelberth J (2009). Monitoring plant hormones during stress responses.. J Vis Exp.

[54] Engelberth J, Alborn HT, Schmelz EA, Tumlinson JH (2004). Airborne signals prime plants against insect herbivore attack.. Proc Natl Acad Sci USA.

